# Genomic characterization of *Salmonella enterica* serovar Kentucky and London recovered from food and human salmonellosis in Zhejiang Province, China (2016–2021)

**DOI:** 10.3389/fmicb.2022.961739

**Published:** 2022-08-04

**Authors:** Lei Fang, Guankai Lin, Yi Li, Qiange Lin, Huihuang Lou, Meifeng Lin, Yuqin Hu, Airong Xie, Qinyi Zhang, Jiancang Zhou, Leyi Zhang

**Affiliations:** ^1^Department of Critical Care Medicine, Sir Run Run Shaw Hospital, College of Medicine, Zhejiang University, Hangzhou, China; ^2^Wenzhou Center for Disease Control and Prevention, Wenzhou, China; ^3^Key Laboratory of Microbial Technology and Bioinformatics of Zhejiang Province, Hangzhou, China; ^4^School of Medical Technology and Information Engineering, Zhejiang Chinese Medical University, Hangzhou, China

**Keywords:** whole-genome sequencing, multidrug resistance, phylogenomic, *Salmonella* Kentucky, *Salmonella* London, food safety

## Abstract

Increasing human salmonellosis caused by *Salmonella enterica* serovar Kentucky and London has raised serious concerns. To better understand possible health risks, insights were provided into specific genetic traits and antimicrobial resistance of 88 representative isolates from human and food sources in Zhejiang Province, China, during 2016–2021. Phylogenomic analysis revealed consistent clustering of isolates into the respective serovar or sequence types, and identified plausible interhost transmission *via* distinct routes. Each serovar exhibited remarkable diversity in host range and disease-causing potential by cgMLST analyses, and approximately half (48.6%, 17/35) of the food isolates were phylogenetically indistinguishable to those of clinical isolates in the same region. *S*. London and *S*. Kentucky harbored serovar-specific virulence genes contributing to their functions in pathogenesis. The overall resistance genotypes correlated with 97.7% sensitivity and 60.2% specificity to the identified phenotypes. Resistance to ciprofloxacin, cefazolin, tetracycline, ampicillin, azithromycin, chloramphenicol, as well as multidrug resistance, was common. High-level dual resistance to ciprofloxacin and cephalosporins in *S*. Kentucky ST198 isolates highlights evolving threats of antibiotic resistance. These findings underscored the necessity for the development of effective strategies to mitigate the risk of food contamination by *Salmonella* host-restricted serovars.

## Introduction

Government regulatory agencies continue to underscore the importance of non-typhoidal *Salmonella* as a leading cause of bacterial foodborne illnesses in humans (Scallan et al., 2011; EFSA ECDC, 2018). Non-typhoidal Salmonellae are zoonotic pathogens those inflict a significant burden on public health in both developing and industrialized countries and cause more than 90 million cases of acute gastroenteritis every year, accounting for ~155,000 deaths worldwide (Kirk et al., 2015; WHO, 2015). In spite of mild and self-limiting nature of human salmonellosis, children and immunocompromised patients with certain underlying conditions have an increased risk of life-threatening diseases and death from its complications (Bula-Rudas et al., 2015).

Most non-typhoidal *Salmonella* infections are attributed to a single species, *Salmonella enterica*, which contains over 2,600 serovars (Kümmerer, 2004). Although these serovars are closely related, they vary in their host ranges and abilities to cause human infections. Human-associated serovars such as *S*. Enteritidis and *S*. Typhimurium display a broad range of host adaptation, whereas *S. enterica* serovar Kentucky and London have a restricted host range (Foley et al., 2008; Haley et al., 2016). *S*. Kentucky is notified by the United States Department of Agriculture as one of the most common serovars isolated from broiler chickens in the United States, comprising 25–51% of all isolates between 1998 and 2013 (Haley et al., 2019). Historically, this serovar has rarely been implicated in human illness and most human cases are generally limited to sequence type 198 (ST198) clonal complex (Le Hello et al., 2011). *S*. London showcases host specificity signature as well and only a few human infections have been reported to be associated with contaminated dairy products in Korea (Kim et al., 2003; Park et al., 2004).

*S. enterica* host-restricted serovars have gained notoriety in recent years as contagious agents of human salmonellosis. This situation has developed from the globalization of food chain that favored their host range expansion by horizontal gene transfer and enhanced their ability to invade new hosts as potential vehicles of human infections on an international scale. *S*. London begins to emerge in various sources (human, pets, wild birds, food animals, and environment) other than dairy-related items and spreads rapidly throughout the world (Bosilevac et al., 2009; Trimoulinard et al., 2017; Xu et al., 2021). In addition to globalization, abuse of antibiotics in animal husbandry plays an important role in the dissemination of multidrug-resistant *Salmonella*, as the transfer of antimicrobial resistance (AMR) genes is easily facilitated by plasmid or transposon exchange between microbial populations (Behzadi et al., 2020, 2021; Woh et al., 2021). In the last 20 years of evolving threat to public health is the global emergence of ciprofloxacin-resistant (CIP^R^) *S*. Kentucky, rendering very limited treatment options (Le Hello et al., 2013a,b; WHO, 2014). According to the survey of European Centers for Disease Control and Prevention, 12 countries reported human salmonellosis caused by CIP^R^ *S*. Kentucky between 2007 and 2012, predominantly in Northern Africa, Europe, and Southern Asia (Westrell et al., 2014).

In China, *S. enterica* is thought to contribute to 70–80% of bacterial foodborne infections, which has a great impact on the Chinese food industry (Shen et al., 2022). However, the population structures of *S. enterica* host-restricted serovars such as *S*. Kentucky and *S*. London in China remain largely unknown. Therefore, the study herein was aimed to investigate the diversity, virulence potential, and antimicrobial resistance of *S*. Kentucky and *S*. London strains recovered from autochthonous human infections and contaminated food products in Zhejiang Province using whole-genome sequencing (WGS). During the course of study, the ability of WGS to predict antimicrobial resistance was evaluated in *S*. Kentucky and *S*. London.

## Materials and methods

### Isolation selection

A total of 62 *S*. London isolates representing human infections (*n* = 41) and food/animal sources (*n* = 21) were involved in this study. To avoid redundancy, isolates were randomly stratified to encompass diverse pulsed field gel electrophoresis (PFGE) profiles, isolation years, sources, sequence types, geographic origins, and AMR phenotypes and genotypes. For *S*. Kentucky, all viable isolates were selected for genomic analysis, including 14 isolates from food samples and 12 isolates from confirmed human salmonellosis during 2016–2021 in Zhejiang Province, China. An elaborated flowchart demonstrating strains from initial sampling to structure characterization was created using the IHMC CmapTools (https://cmap.ihmc.us, Supplementary Figure 1; Behzadi and Gajdács, 2021) and the details of these strains are presented in Supplementary Table 1.

### Genome library preparation and whole-genome sequencing

Genomic DNA was obtained from a single colony picked from trypticase soy agar using a plant Genomic DNA kit (Tiangen, DP305) according to the manufacturer's instructions. Extracted DNA for each isolate was quantified using a NanoDrop^TM^ 2000 (Thermo Fisher Scientific, Waltham, MA, USA) spectrophotometer and verified by agarose gel electrophoresis (Supplementary Figure 2). Extracted DNA with concentration ≥100 ng was selected and sent to a professional sequencing company (Hangzhou Digital-Micro Biotech Co., Ltd) for genomic library preparation. Sequencing libraries were prepared using the TruePrep^TM^ DNA library preparation kit (Vazyme, China) for Illumina following manufacturer's instructions. Using a single “transposase” enzymatic reaction, DNA sample was simultaneously fragmented and tagged with adapters, followed by an optimized, limited-cycle PCR protocol to amplify tagged DNA. Individual library was assessed on the QIAxcel Advanced Automatic nucleic acid analyzer and amplified through qPCR using KAPA SYBR^®^ FAST qPCR Kits (Supplementary Figure 3). Genomic libraries were sequenced on an Illumina Novaseq 6000 platform (Illumina Inc., San Diego, CA, USA) to generate 150-bp paired-end reads. Sequencing and library construction were performed by technical staff at Hangzhou Digital-Micro Biotech Co., Ltd.

### Sequences analyses

FastQC version 0.11.2 was used to confirm that all adapter sequences were removed and all the reads were quality controlled. Raw reads were trimmed by using Trimmomatic version 0.36 and *de novo* genome assemblies were generated using Unicycler (Bolger et al., 2014). Sequence types (STs) were determined from the *Salmonella* MLST database (https://pubmlst.org/salmonella) for assignment of allele identification. Core-genome MLST (cgMLST) assignment based on 3,002 core genes was analyzed with BioNumerics software version 7.6 (Applied Maths, Belgium; Alikhan et al., 2018) and core-genome comparison was visualized using GView Server (https://server.gview.ca/; Behzadi et al., 2016; Ranjbar et al., 2016). The cgMLST profiles were subsequently downloaded to MEGA 7.0 to generate a phylogenetic tree by maximum-likelihood algorithm with 1,000 bootstrap samples. The resulting dendrogram was created in Interactive Tree of Life software (iTOL) (https://itol.embl.de/) version 5.7 (Letunic and Bork, 2016). Notably, 7,346 genes were identified with a pan-genome analysis of 88 *Salmonella* strains using Roary software. These genes were used to perform the principal component analysis (PCA) and the strains were colored on the basis of their sequence types.

To investigate putative virulence genes, sequences with those of the known virulence genes were compared through the Virulence Factor Database (VFDB; http://www.mgc.ac.cn/VFs/) for *Salmonella*, resulting in a comprehensive data set containing 220 genes associated with *Salmonella* virulence factors (Supplementary Table 2; Chen et al., 2005). ABRicate software (https://github.com/tseemann/abricate) was used to compare the genomes of 88 isolates with VFDB database. When the similarity and coverage of the sequence was greater than or equal to 80%, the existence of the virulence gene was studied, which was counted as 1; otherwise, the absence of the virulence gene was considered and counted as 0. Meanwhile, the presence of known antimicrobial resistance genes was mapped against chromosomal and plasmid-resistant genes found in Comprehensive Antibiotic Resistance Database (CARD; https://card.mcmaster.ca/) in conjunction with the Center for Genomic Epidemiology pipeline (CGE; http://www.genomicepidemiology.org/; Jia et al., 2017). Resistance determinants with a minimum coverage of 90% and a minimum identity of 90% were identified.

### Phenotypic antimicrobial resistance testing

The antimicrobial susceptibility of 88 *Salmonella* strains was determined using dehydrated panel CHN1GOVF and CNBGOV2F (Thermo Fisher Scientific, Waltham, MA) against 22 antibiotics. Results were analyzed by the BioNumeric 7.6 software and interpreted as sensitive or resistant using Clinical and Laboratory Standards Institute (CLSI) criteria. The 22 antimicrobials and breakpoints (μg/ml) for determining resistances were as follows: ampicillin (AMP, ≥32), ampicillin-sulbactam (AMS, ≥32/16), amoxicillin-clavulanic acid (AMC, ≥32/16), cefazolin (CFZ, ≥8), cefepime (FEP, ≥16), cefotaxime sodium (CTX, ≥4), cefoxitin (CFX, ≥32), ceftazidime (CAZ, ≥16), azithromycin (AZI, ≥32), imipenem (IMI, ≥4), meropenem (MEM, ≥4), gentamicin (GEN, ≥16), amikacin (AMI, ≥64), kanamycin (KAN, ≥64), tetracycline (TET, ≥16), doxycycline (DOX, ≥16), minocycline (MIN, ≥16), ciprofloxacin (CIP, ≥1), levofloxacin (LEV, ≥2), nalidixic acid (NAL, ≥32), chloramphenicol (CHL, ≥32), and trimethoprim-sulfamethoxazole (SXT, ≥4/76). *Escherichia coli* ATCC 25922 was used as a control organism. Isolates exhibiting resistant patterns to three or more groups of antibiotics were considered as multidrug-resistant (MDR) isolates.

### Statistical analysis

The statistical software SPSS (Statistics 20, IBM, Armonk, NY, USA) was employed for data processing and statistical analysis. A zero-inflated negative binomial regression model was also employed to test for statistically significant associations between sequenced food isolates and sources. This model was also used to determine salmonellosis correlations with the gender and age in humans. Unpaired *t*-test was performed to analyze for differences between contained virulence genes and sequence types as well as significant associations between resistance to different antimicrobial agents, different serovars, and *Salmonella* isolates from different hosts. *P*-values of < 0.05 were considered significant.

## Results

### Phylogenetic analysis of *S. enterica* serovar Kentucky and London isolates from different sources

**Figure 1:**
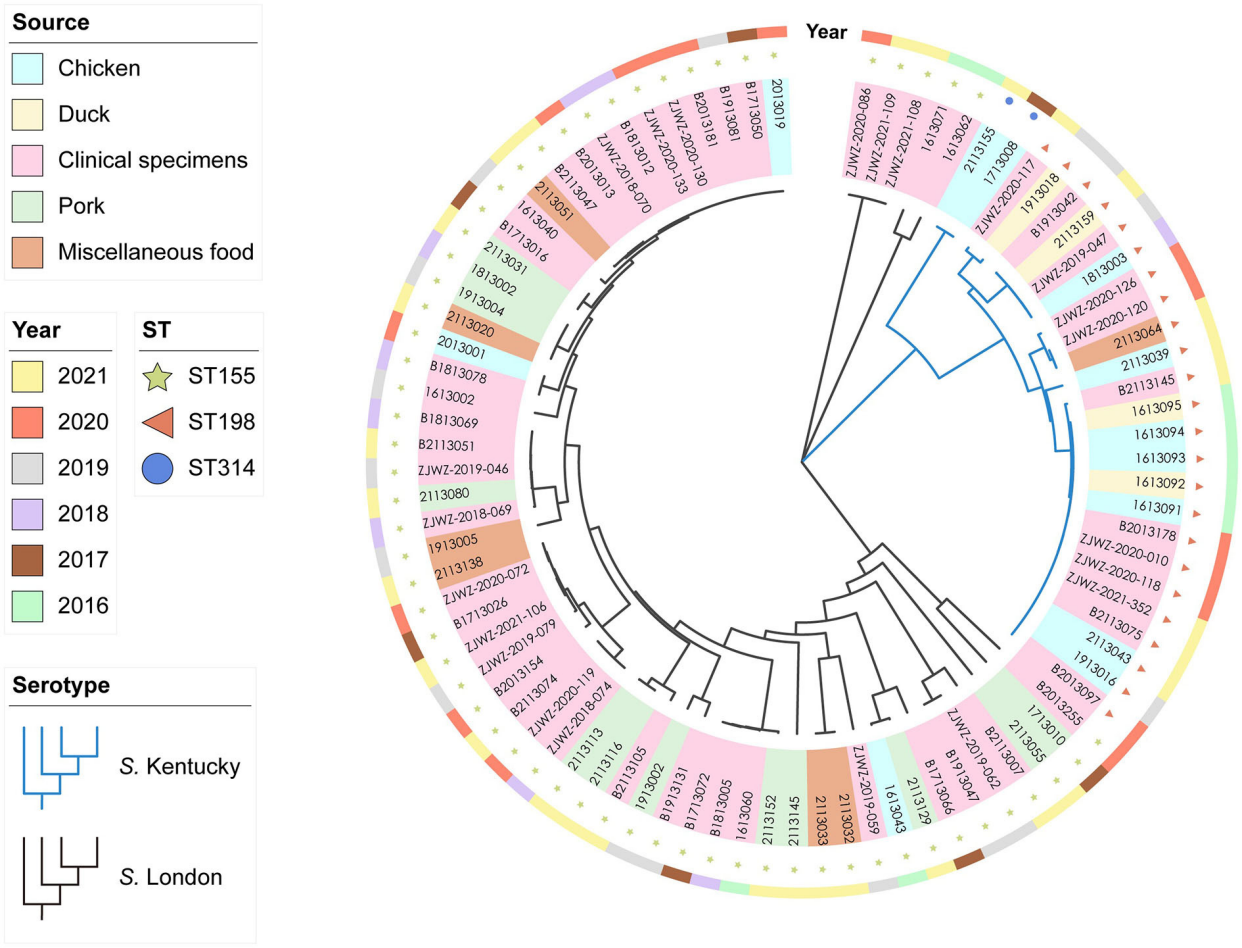
Whole-genome phylogenetic relationships within 88 *Salmonella enterica* serovar Kentucky (*n* = 26) and London (*n* = 62) isolates by cgMLST analysis (~4.5 Mb). The tree was inferred by using the iTOL interactive user interface (https://itol.embl.de). Shading over tip labels indicate sources. Sequence types (STs), including ST155 (*n* = 62), ST198 (*n* = 24), and ST314 (*n* = 2), are annotated by colors and shapes.

Notably, 88 genomes of *S*. Kentucky (*n* = 26) and *S*. London (*n* = 62) were sequenced and underwent cgMLST analysis. No significant association was noted between sequenced food isolates and source (*P* > 0.05). A similar situation was observed in sequenced clinical isolates, as salmonellosis did not correlate with age or gender in humans (*P* > 0.05), suggesting that sequenced isolates were relatively unbiased by putative variables. The phylogenetic tree delineated two distinct clades corresponding to the serovars, demonstrating significant differences of the population structures between *S*. London and *S*. Kentucky isolates (Figure 1). All *S*. London isolates were recognized as ST155 (*n* = 62) and overlaid onto two divergent clusters. *S*. Kentucky clade was formed by two sister clusters representing ST314 (*n* = 2) and ST198 (*n* = 24) isolates, respectively. Clinical isolates were frequently matched isolates from local poultry, pork, or miscellaneous food, corresponding to 56.6% (30/53) of all clinical isolates in the study. For example, six clinical isolates in one small *S*. Kentucky cluster matched five isolates from chicken and two isolates from duck. Seven *S*. London isolates from patients were indistinguishable to one strain from chicken, and formed a sister group with a strain from dumplings (in the category of miscellaneous food). ST155 clinical isolates (36.6%, 15/41) clustered most often with the isolates of pork origin, while ST198 clinical isolates were common for clustering isolates from chicken and duck hosts (Supplementary Figure 4). Despite high-level diversity of the dataset in general, 85.7% (36/41) of ST155 clinical isolates showed close association with each other and formed one large genetic group. Moreover, 75% (9/12) of ST198 clinical isolates collected from different sampling years were confined to the same cluster.

### Molecular characteristics associated with virulence

**Figure 2:**
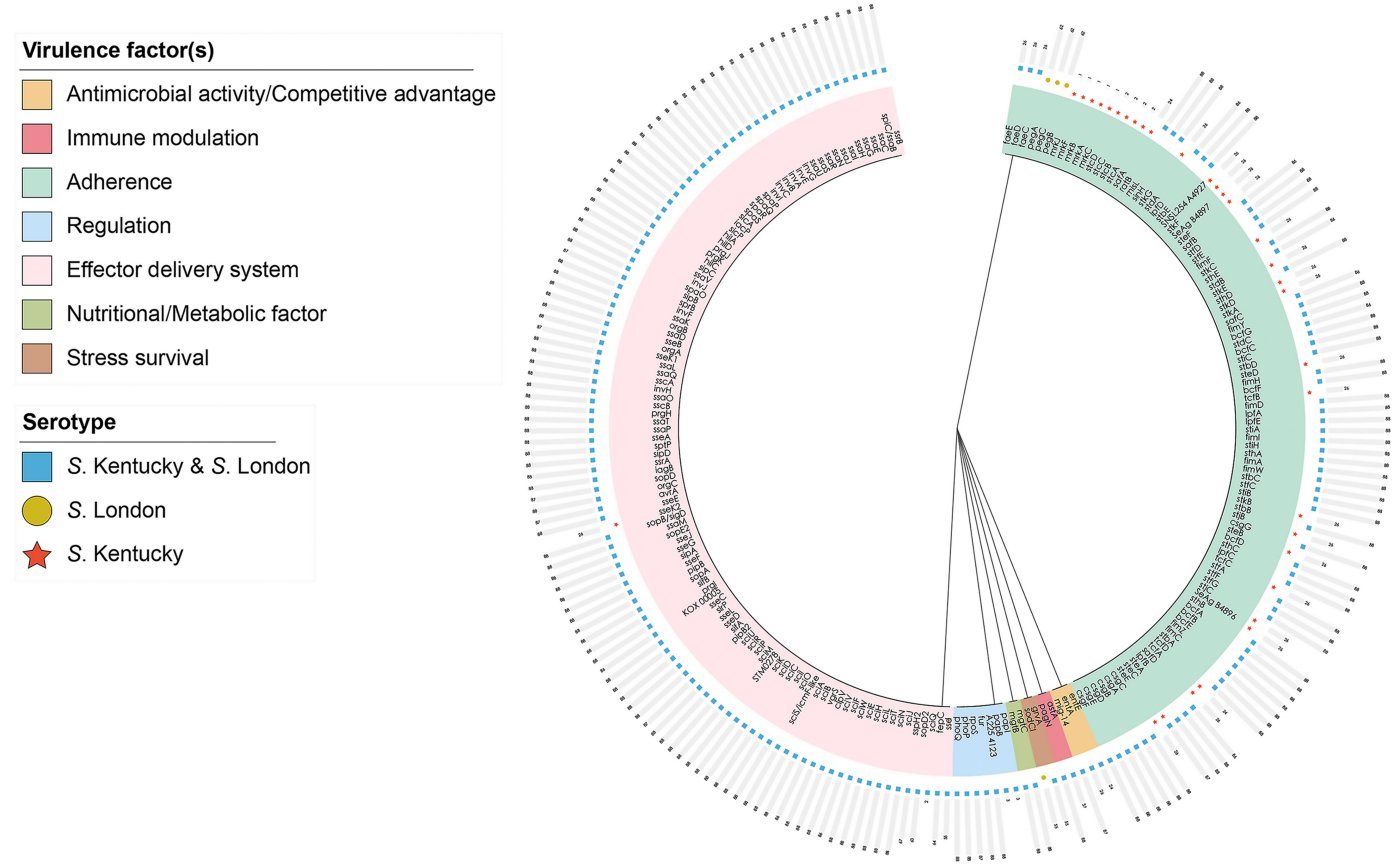
Distribution of 220 putative virulence genes harbored by *Salmonella enterica* serovar Kentucky and London isolates in Zhejiang, 2016–2021. The hierarchically clustered circular tree was inferred by using the iTOL interactive user interface (https://itol.embl.de). Shading over tip labels indicate gene functions. Serotypes are annotated by colors and shapes.

**Figure 3:**
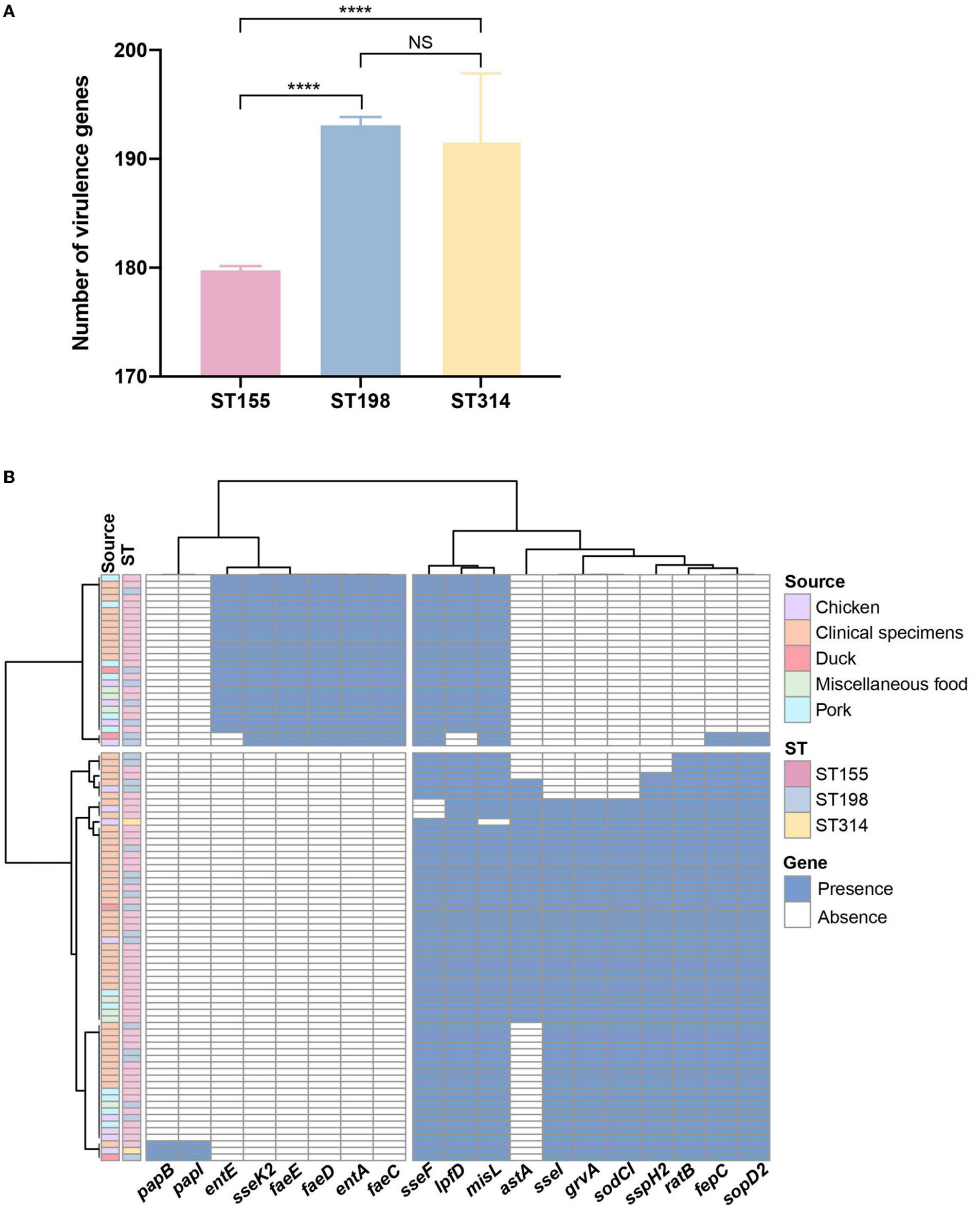
Genome analysis of putative virulence genes among *Salmonella enterica* serovar Kentucky and London isolates. **(A)** Abundance of putative virulence genes identified among different sequence types. Error bars indicate mean with 95% CL. **(B)** Distribution of putative virulence genes showed discrepancies within 88 *Salmonella* isolates in the PCA-based prediction model. The blue color in the cell indicates the presence of virulence genes and the white color indicates the corresponding absence of virulence genes. Sequence types and sources are annotated by colors. *NS*, no significance; ****P < 0.0001.

After comparison with the known virulence genes in virulence factor database, a total of 220 putative virulence genes were detected *in silico* and details of these virulence genes are presented in Supplementary Table 2. Putative virulence genes were classified into seven functional groups, including adherence, antimicrobial activity, effector delivery system, immune modulation, nutritional/metabolic factor, regulation, and stress survival (Figure 2). Notably, 84.1% of the genes (185/220) were highly conserved and possessed by both *S*. London and *S*. Kentucky isolates. In total, 31 virulence genes encoding for adherence and one virulence gene contributing to the bacterial delivery system were found exclusively in *S*. Kentucky isolates. Three virulence genes involved in the process of *Salmonella* attachment were only associated with *S*. London isolates. Although a significant difference (*P* < 0.0001) in total virulence genes was observed between ST155 and ST198/ST314 isolates at the pan-genomic level (Figure 3A), there was confounding for specific virulence genes, which were not well-distinguished (Figure 3B).

### Antibiotic susceptibility analysis

To investigate bacterial resistance patterns, antimicrobial susceptibility tests were performed against 22 antimicrobials belonging to 10 classes or categories, and the frequency of AMR results is presented in Table 1. The most common resistances for *S*. Kentucky isolates were to ciprofloxacin, tetracycline, doxycycline, and nalidixic acid (92.3%; *n* = 24). The AMR rate was significantly higher in *S*. Kentucky than that in *S*. London strains (*P* < 0.001). Among the *S*. London isolates, resistance to doxycycline (74.2%; *n* = 46) was the most frequent, followed by tetracycline (71%; *n* = 44), ampicillin (64.5%; *n* = 40), and gentamicin (56.5%; *n* = 35). Low-level resistance to imipenem and meropenem was detected for both *S*. London and *S*. Kentucky isolates.

**Table 1:** Antimicrobial resistant rate of *Salmonella enterica* serovar Kentucky and London isolates against 22 antimicrobials.

**Antimicrobial class**	**Antibiotic (breakpoints, μg/mL)**	**Resistant rate (%) of isolates by serovar**		**Resistant rate (%) of isolates by species**	
		***Salmonella* Kentucky (*n* = 26)**	***Salmonella* London (*n* = 62)**	**Food (*n* = 35)**	**Human (*n* = 53)**
Penicillin	AMP (≥32)	88.5	64.5	54.3	83.0
Beta-lactam/beta-lactam inhibitor	AMS (≥32/16)	53.8	37.1	51.4	52.8
	AMC (≥32/16)	11.5	12.9	0.0	20.8
Cephalosporins	CFZ (≥8)	80.7	37.1	31.4	66.0
	FEP (≥16)	26.9	6.5	5.7	17.0
	CTX (≥4)	61.5	8.1	17.1	28.3
	CFX (≥32)	11.5	6.5	0.0	13.2
	CAZ (≥16)	57.7	6.5	4.0	28.3
Macrolide	AZI (≥32)	57.7	8.1	34.3	32.1
Carbapenems	IMI (≥4)	3.8	6.5	0.0	9.4
	MEM (≥4)	3.8	4.8	0.0	7.5
Aminoglycosides	GEN (≥16)	76.9	56.5	54.3	67.9
	AMI (≥64)	23.1	1.6	8.6	7.5
	KAN (≥64)	84.6	22.6	34.3	45.3
Tetracycline	TET (≥16)	92.3	71.0	62.9	92.5
	DOX (≥16)	92.3	74.2	62.9	92.5
	MIN (≥16)	80.8	2.7	25.8	54.7
Quinolone	CIP (≥1)	92.3	38.7	54.3	54.7
	LEV (≥2)	88.5	11.3	34.3	34.0
	NAL (≥32)	92.3	16.1	34.3	41.5
Phenicol	CHL (≥32)	80.8	67.7	62.9	77.4
Sulfonamides	SXT (≥4/76)	69.2	56.5	51.4	66.0

**Figure 4:**
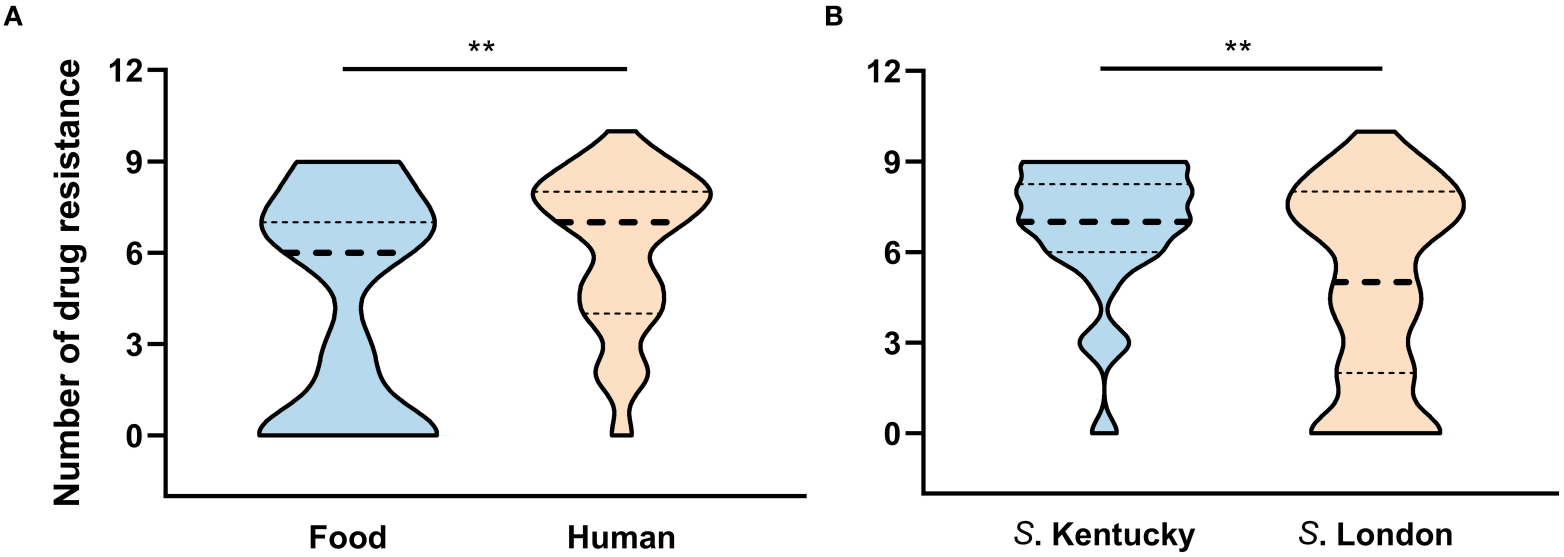
The number of antimicrobial resistance patterns classified by **(A)** source and **(B)** serovar. **P < 0.01.

Among the isolates characterized, 84.1% (*n* = 74) were resistant to at least one of the tested antimicrobial compounds and 52.3% (*n* = 46) revealed MDR profiles. In total, 44% of the isolates (*n* = 39) exhibited resistance to at least five classes of antibiotics, and 35.2% (*n* = 31) were resistant to at least 7 of the 10 classes tested. As shown in Figure 4, representative human isolates exhibited a significantly higher rate of MDR (*P* = 0.0057) than those isolates collected from food sources. Human and food strains showed median resistance to seven and six classes of antimicrobials, respectively. Notably, one strain that showed resistance to 10 classes of antibiotics was collected from human fecal sample.

Considering resistance by serovar, 96.2% (25/26) of isolates belonging to serovar Kentucky exhibited MDR and 84.6% (22/26) of these MDR isolates were resistant to over six classes of the 10 tested antibiotics (Figure 4). Although one *S*. London strain exhibited resistance to 10 classes of tested agents, the MDR rate was significant lower in *S*. London relative to *S*. Kentucky strains (*P* = 0.0041). Notably, 67.7% (42/62) of *S*. London isolates exhibited MDR and displayed median resistance to five classes of antibiotics. When separation was done based on ST assignment, two *S*. Kentucky isolates assigned to ST314 were distinct from the remaining ST198 strains, which were all MDR. One ST314 strain had no resistance to any of the antibiotics and the other one was resistant to three classes of the tested antimicrobial compounds. All *S*. London isolates were assigned to ST155 and had significant lower MDR rate compared with ST198 isolates (*P* < 0.01).

### Correlation of antimicrobial susceptibility phenotypes and genotypes

The 88 *Salmonella* isolates were further subjected to *in silico* detection of antimicrobial resistance genes. A total of 86 unique genes encoding resistance to 10 antimicrobial classes were detected. The identified resistance genes were evaluated of their ability to predict phenotypic resistance (Table 2). Penicillin, cephems, and carbapenems were not included in the analysis due to the rarity of resistance genes identified. For the remaining seven antimicrobial classes, *in silico* AMR gene detection was highly correlated with phenotypic AMR patterns, resulting in an overall sensitivity of 97.7% and a specificity of 60.2%. Genotypic prediction of phenotypic resistance to aminoglycosides, beta-lactam/beta-lactam inhibitor, macrolide, phenicol, and sulfonamides had a sensitivity of 100%, while the prediction of phenotypic resistance to beta-lactam/beta-lactam inhibitor and tetracycline yielded a specificity of 100%. With the exception of quinolone, genotypic prediction of phenotypic resistance resulted in sensitivities >90% for the remaining six antimicrobial classes. Aminoglycosides, macrolide, quinolone, and sulfonamides presented poor coherence between phenotypic resistance and the presence of corresponding resistance genes, yielding specificities <60%.

**Table 2:** Genotype and phenotype comparison of *Salmonella enterica* serovar Kentucky and London isolates from food and humans, 2016 to 2021.

**Antibiotics**	**Phenotype: resistant**		**Phenotype: susceptible**		**Sensitivity (%)**	**Specificity (%)**
	**Genotype: resistant**	**Genotype: susceptible**	**Genotype: resistant**	**Genotype: susceptible**		
Aminoglycosides	58	0	30	0	100	0
Beta-lactam/beta-lactam inhibitor	63	0	0	25	100	100
Macrolide	29	0	25	34	100	57.6
Phenicol	63	0	4	21	100	84.0
Quinolone	45	8	10	25	84.9	1.6
Sulfonamides	53	0	21	14	100	40.0
Tetracycline	70	1	0	17	98.6	100
Total	381	9	90	136	97.7	60.2

## Discussion

*S. enterica* host-restricted serovars have been increasingly recognized as suspected etiological agents for foodborne diseases (Li et al., 2014; Xu et al., 2021). As emerging serovars, *S*. Kentucky and *S*. London have not been well-elucidated in China. Thus, the information on the diversity of *S*. Kentucky and *S*. London isolates from food and human salmonellosis can bridge the knowledge in Zhejiang Province and provide a reference for other areas in China. The phylogenetic tree was diversified into three clusters corresponding to the serovar and sequence type, confirming the genetic homogeneity of the isolates from the same serovar/ST. Human infections caused by *S*. Kentucky ST198 were not related to travel abroad, as many clinical isolates were genetically clonal to the strains isolated from local chicken and ducks, suggesting a spatiotemporal persistence of *S*. Kentucky ST198 in the Zhejiang area. This finding was inconsistent to the situation in Northern America and Europe, where more than half of the ST198 human cases were associated with traveling to the areas of ST198 endemicity (Westrell et al., 2014; Haley et al., 2019). As expected, *S*. London genomes exhibited remarkable diversity, in line with the increasing understanding of *S*. London distribution, which was formed by a high degree of horizontal gene transfer (Shipp and Dickson, 2011; Trimoulinard et al., 2017). Close associations between humans and food animals (pork, chicken, beef, and animal-derived food products in the category of miscellaneous food) were previously noted and were reaffirmed by the present survey. It is noteworthy that one strain recovered from lettuce (strain ID: 2113020, in the category of miscellaneous food) showed close relatedness to clinical isolates, indicating that *S*. London related salmonellosis was likely linked to the contaminated vegetables. Despite the fact that *S*. London has been reported with a restricted host range, transmission of this serovar from a zoonotic source to a non-animal food vehicle is possible. *S*. London carried by domesticated and wild animals can contaminate fresh produce by direct or indirect contact (Hanning et al., 2009; Amponsah-Doku et al., 2010). This occasional detection may reflect inadequate control measures, causing cross-contamination of *S*. London in lettuce. As clinical isolates did not correlate with the age in this study, salmonellosis may disproportionately affect young children and immunocompromised patients when they consumed contaminated animal meats or fresh produce. Therefore, these findings underscore the necessity for the development of food safety technologies to minimize the risk of *Salmonella* outbreaks in the Zhejiang area.

Detailed investigation into the pathogenicity revealed that *S*. Kentucky (ST198 and ST314) isolates possessed more putative virulence genes than *S*. London (ST155) isolates, suggesting that *S*. Kentucky might have higher virulence potential than *S*. London in general. This finding was in agreement with the reports in other countries and regions, where *S*. London was typically responsible for asymptomatic or mild human infections, but *S*. Kentucky could cause disease with a significant consequence (Westrell et al., 2014; Coipan et al., 2020; Xu et al., 2021). Both *S*. London and *S*. Kentucky harbored serovar-specific virulence genes. The *sseK2* gene encoding novel translocated protein was only present in serovar Kentucky, which may increase the ability of *S*. Kentucky for biofilm formation and proliferation in extreme environments (Kujat Choy et al., 2004; Zhang et al., 2019). Meanwhile, the genes encoding major subunits of SEF14 fimbriae (*pegA, pegB*, and *pegC*) were sporadically detected in *S*. London but not in *S*. Kentucky strains in this study. The expression of SEF14 fimbriae has only been reported by *S*. Enteritidis and its closely related serovars, which was essential for these serovars to adhere and colonize in poultry gut (Turcotte and Woodward, 1993; Edwards et al., 2000). Thus, the *pegABC*-encoding fimbriae may confer serovar advantage for *S*. London to survive in poultry host (Clayton et al., 2008). To be noted, human and food isolates contained indistinguishable virulence genes (*P* > 0.05), indicating a fascinating mixture of genetic elements among different sources. Variable host adaptation between *S*. London and *S*. Kentucky strains might be attributed to the differential conservation of adherent components/effectors or colonization factors. Therefore, deciphering the colonization requirements among different serovars in future studies will be a useful endeavor to understand *Salmonella* host adaptation.

The rise in antimicrobial resistance continues to be a worldwide crisis. Before the 1960's, *S. enterica* was susceptible to all antibiotics. The appearance of *S*. Kentucky ST198 strains in the 1990's raised major concerns because of its high-level resistance to ciprofloxacin (Le Hello et al., 2012; Hawkey et al., 2019). It is worth mentioning that 92.3% (24/26) of *S*. Kentucky from human and food samples were resistant to ciprofloxacin in this study. Only two isolates assigned to ST314 showed sensitivity to ciprofloxacin, while all ST198 isolates were resistant to ciprofloxacin, which was substantially higher than the previously reported CIP^r^ *S*. Kentucky in China or many other countries (Haley et al., 2019; Hawkey et al., 2019; Xiong et al., 2020). In addition, up to 87.5% (21/24) of ST198 isolates were further resistant to extended-spectrum cephalosporins (ESCs). Ciprofloxacin and ESCs are used most frequently in chemotherapy for invasive salmonellosis. Dual resistance to ciprofloxacin and third-generation cephalosporins poses great threats to human health, as treatment failure could have severe outcome (Le Hello et al., 2013b). The persistence of dual-resistant clone in Zhejiang marks a sentinel event in the evolution of antibiotic resistance in *S*. Kentucky. Although *S*. London isolates exhibited reduced resistance to ciprofloxacin and cephalosporins, high-level resistance to tetracycline and ampicillin was common and several strains displayed resistance to the last-line oral antibiotic azithromycin. These results differed from a *S*. London report of Spanish environmental isolates showing sensitive patterns to tetracycline and ampicillin (Espigares et al., 2006). These observations also contrasted with a 2009 survey of ground meat in the United States, whereby *S*. London was found to be sensitive to tetracycline but resistant to ampicillin (Bosilevac et al., 2009). The overall rise in resistance to these antimicrobial agents reflected genetic reassortment in *S*. London (Huber et al., 2021; Shen et al., 2022). Moreover, the high prevalence of MDR strains in Zhejiang provided evidence of anthropogenic impact on *S. enterica* host-restricted serovars, leading to limited drug of choices to treat indigenous Salmonella infections.

Diagnostic tests using DNA sequences (dry laboratory, *in silico*) and other culture-independent methods (wet laboratory, *in vitro*) have seen accuracy in identifying bacterial species and the source of outbreaks (Langley et al., 2015; Ranjbar et al., 2017). WGS has proven to be effective in predicting antimicrobial susceptibility and is becoming common as part of a routine laboratory workflow in western countries (Stoesser et al., 2013; Gordon et al., 2014; Tyson et al., 2015). As few studies regarding this technique have been evaluated in China, the power of WGS was explored to identify antimicrobial resistance in salmonellae during the course of study. It was found that resistance genes detected *in silico* showed high-level concordance to phenotypic profiles and this observation was consistent with the study by Zhao et al., who examined 640 strains of non-typhoidal *Salmonella* from humans and retail meats using WGS and found an overall correlation of 99% (McDermott et al., 2016). In addition, a prior study of 50 *Salmonella* strains from swine in Denmark found complete agreement between phenotypic and genotypic resistance (Zankari et al., 2013). Hence, these results support the hypothesis that WGS is a powerful tool for AMR prediction in non-typhoidal *Salmonella*.

## Conclusion

In an era of globalized food systems and increasing foodborne outbreaks caused by *S. enterica*, this study served as a compelling example to understand genetic diversity and antimicrobial resistance of *S. enterica* host-restricted serovars. The cgMLST analyses demonstrated that a significant proportion of *S*. London and *S*. Kentucky isolates from various food sources were identical to the strains from clinical settings. It is worth mentioning that CIP^r^ *S*. Kentucky and MDR *S*. London were highly prevalent in food animals in Zhejiang. These findings strengthened the argument for close monitoring of autochthonous *S. enterica* populations, especially those with host specificity signatures. These data may be useful for regulatory agencies and food safety communities to develop intervention strategies to constrain non-typhoidal *Salmonella* infections.

## Data availability statement

The datasets presented in this study can be found in online repositories. The names of the repository/repositories and accession number(s) can be found in the article/Supplementary material.

## Ethics statement

The studies involving human participants were reviewed and approved by Zhejiang University. The patients/participants provided their written informed consent to participate in this study.

## Author contributions

LZ, YL, and LF conceived and designed the study. ML, HL, YH, and AX performed the sampling. LF, QZ, and LZ analyzed WGS data. LF and LZ prepared the manuscript draft and revised the manuscript. JZ and GL performed the administration. JZ, GL, QL, and ML contributed reagents and materials and analysis tools. All authors contributed to the article and approved the submitted version.

## Funding

This study was funded by Wenzhou Science Research Projects (Y20180202).

## Conflict of interest

The authors declare that the research was conducted in the absence of any commercial or financial relationships that could be construed as a potential conflict of interest.

## Publisher's note

All claims expressed in this article are solely those of the authors and do not necessarily represent those of their affiliated organizations, or those of the publisher, the editors and the reviewers. Any product that may be evaluated in this article, or claim that may be made by its manufacturer, is not guaranteed or endorsed by the publisher.
