# Supplementary material for: Genomic characterization of Salmonella enterica serovar Kentucky and London recovered from food and human salmonellosis in Zhejiang Province, China (2016–2021)
Source: Front Microbiol. 2022 Aug 4;13:961739. doi: 10.3389/fmicb.2022.961739 (PMC9437622; doi:10.3389/fmicb.2022.961739)
Supplement: Supplementary file 1 [file Data_Sheet_1.pdf]

Supplementary Table S1 Summary of *Salmonella* Kentucky and London strains

Strain ID	Serotype	Source	Year and Month	Sequence Type	Sample Type
1613002	London	Human	2016.04	155	[1]Feces (Male, 65)
1613040	London	Human	2016.08	155	Feces (Female, 34)
1613060	London	Human	2016.10	155	Feces (Male, 74)
1613062	London	Human	2016.10	155	Feces (Male, 59)
1613071	London	Human	2016.11	155	Feces (Male, 51)
B1713016	London	Human	2017.06	155	Feces (Male, 64)
B1713026	London	Human	2017.07	155	Feces (Male, 23)
B1713050	London	Human	2017.08	155	Feces (Female, 67)
B1713066	London	Human	2017.09	155	Feces (Female, 27)
B1713072	London	Human	2017.09	155	Feces (Female, 39)
B1813005	London	Human	2018.01	155	Feces (Male, 37)
B1813012	London	Human	2018.05	155	Feces (Female, 31)
B1813069	London	Human	2018.08	155	Feces (Male, 53)
B1813078	London	Human	2018.09	155	Feces (Male, 37)
B1913047	London	Human	2019.07	155	Feces (Male, 59)
B1913081	London	Human	2019.08	155	Feces (Male, 62)
B1913131	London	Human	2019.10	155	Feces (Male, < 1)
B2013013	London	Human	2020.05	155	Feces (Male, 14)
B2013154	London	Human	2020.08	155	Feces (Male, 48)
B2013181	London	Human	2020.09	155	Feces (Male, 27)
B2013255	London	Human	2020.11	155	Feces (Female, 65)
B2113007	London	Human	2021.02	155	Feces (Male, 32)
B2113047	London	Human	2021.02	155	Feces (Female, 62)
B2113051	London	Human	2021.06	155	Feces (Female, 44)
B2113074	London	Human	2021.07	155	Feces (Male, 51)
B2113105	London	Human	2021.07	155	Feces (Male, 27)
ZJWZ-2018-069	London	Human	2018.06	155	Feces (Male, 13)
ZJWZ-2018-070	London	Human	2018.05	155	Feces (Male, 50)
ZJWZ-2018-074	London	Human	2018.08	155	Feces (Female, 53)
ZJWZ-2019-046	London	Human	2019.04	155	Feces (Male, 21)
ZJWZ-2019-059	London	Human	2019.04	155	Feces (Female, 26)
ZJWZ-2019-062	London	Human	2019.05	155	Feces (Male, 30)
ZJWZ-2019-079	London	Human	2019.07	155	Feces (Female, 4)
ZJWZ-2020-072	London	Human	2020.06	155	Feces (Male, 10)
ZJWZ-2020-086	London	Human	2020.07	155	Feces (Female, < 1)
ZJWZ-2020-119	London	Human	2020.07	155	Feces (Female, 83)
ZJWZ-2020-130	London	Human	2020.05	155	Feces (Female, 69)
ZJWZ-2020-133	London	Human	2020.08	155	Blood (Male, 61)

Continued

Strain ID	Serotype	Source	Year and Month	Sequence Type	Sample Type
ZJWZ-2021-106	London	Human	2021.06	155	Feces (Male, < 1)
ZJWZ-2021-108	London	Human	2021.06	155	Feces (Male, 38)
ZJWZ-2021-109	London	Human	2021.06	155	Urine (Female, 73)
B1913042	Kentucky	Human	2019.07	198	Feces (Male, 72)
B2013097	Kentucky	Human	2020.06	198	Feces (Female, 54)
B2013178	Kentucky	Human	2020.09	198	Feces (Male, < 1)
B2113075	Kentucky	Human	2021.07	198	Feces (Male, 36)
B2113145	Kentucky	Human	2021.09	198	Feces (Female, 48)
ZJWZ-2019-047	Kentucky	Human	2019.03	198	Feces (Female, 16)
ZJWZ-2020-010	Kentucky	Human	2020.01	198	Feces (Female, 26)
ZJWZ-2020-117	Kentucky	Human	2020.07	198	Feces (Male, 45)
ZJWZ-2020-118	Kentucky	Human	2020.07	198	Feces (Male, 1)
ZJWZ-2020-120	Kentucky	Human	2020.08	198	Feces (Male, 37)
ZJWZ-2020-126	Kentucky	Human	2020.09	198	Feces (Male, 33)
ZJWZ-2021-352	Kentucky	Human	2021.07	198	Feces (Male, 45)
1613043	London	Food	2016.08	155	Chicken
1713010	London	Food	2017.09	155	Pork
1813002	London	Food	2018.07	155	Pork
1913002	London	Food	2019.04	155	Pork
1913004	London	Food	2019.07	155	Pork
1913005	London	Food	2019.07	155	[2]MF (Beef)
2013001	London	Food	2020.05	155	Chicken
2013019	London	Food	2020.11	155	Chicken
2113020	London	Food	2021.04	155	MF (Lettuce)
2113031	London	Food	2021.04	155	Pork
2113032	London	Food	2021.05	155	MF (Cold rice noodle)
2113033	London	Food	2021.05	155	MF (Bean jelly)
2113051	London	Food	2021.07	155	MF (Dumpling)
2113055	London	Food	2021.07	155	Pork
2113080	London	Food	2021.07	155	Pork
2113113	London	Food	2021.07	155	Pork
2113116	London	Food	2021.07	155	Pork
2113129	London	Food	2021.08	155	Pork
2113138	London	Food	2021.09	155	MF (Wonton)
2113145	London	Food	2021.10	155	Pork
2113152	London	Food	2021.10	155	Pork
1613091	Kentucky	Food	2016.08	198	Chicken
1613092	Kentucky	Food	2016.08	198	Duck
1613093	Kentucky	Food	2016.10	198	Chicken
1613094	Kentucky	Food	2016.10	198	Chicken
1613095	Kentucky	Food	2016.10	198	Duck

Continued

Strain ID	Serotype	Source	Year and Month	Sequence Type	Sample Type
1713008	Kentucky	Food	2017.09	314	Chicken
1813003	Kentucky	Food	2018.07	198	Chicken
1913016	Kentucky	Food	2019.12	198	Chicken
1913018	Kentucky	Food	2019.12	198	Duck
2113039	Kentucky	Food	2021.06	198	Chicken
2113043	Kentucky	Food	2021.06	198	Chicken
2113064	Kentucky	Food	2021.07	198	MF (Beef)
2113155	Kentucky	Food	2021.11	314	Chicken
2113159	Kentucky	Food	2021.11	198	Duck

[1] Clinical sample type includes gender and age of the patients with salmonellosis

[2] MF = Miscellaneous food; Miscellaneous food includes beef, wonton, dumpling, bean jelly, cold rice noodle and lettuce.

Supplementary Table S2 Virulence gene detections based on WGS in *Salmonella* Kentucky and London strains

Virulence gene	Gene function	No. of isolated strains
csgF	Adherence	88
csgE	Adherence	88
csgD	Adherence	88
csgB	Adherence	88
csgA	Adherence	88
csgC	Adherence	88
steE	Adherence	26
steC	Adherence	87
steA	Adherence	88
lpfB	Adherence	88
safD	Adherence	86
tcfA	Adherence	26
tcfD	Adherence	24
stbA	Adherence	88
fimC	Adherence	88
fimZ	Adherence	88
bcfE	Adherence	88
bcfB	Adherence	88
bcfA	Adherence	88
sthB	Adherence	88
SeAg_B4896	Adherence	26
stjC	Adherence	26
stfG	Adherence	88
stfF	Adherence	88
stfA	Adherence	88
tcfC	Adherence	26
lpfC	Adherence	88
sthC	Adherence	88
bcfD	Adherence	88
steB	Adherence	26
csgG	Adherence	88
stjB	Adherence	26
stbB	Adherence	88
stkB	Adherence	26
stiB	Adherence	88
stfC	Adherence	88
stbC	Adherence	88
fimW	Adherence	88

Continued

Virulence gene	Gene function	No. of isolated strains
fimA	Adherence	88
sthA	Adherence	88
stiH	Adherence	88
fimI	Adherence	88
stiA	Adherence	88
lpfE	Adherence	88
lpfA	Adherence	88
fimD	Adherence	88
tcfB	Adherence	26
bcfF	Adherence	88
fimH	Adherence	88
steD	Adherence	26
stbD	Adherence	88
stiC	Adherence	88
bcfC	Adherence	88
stdC	Adherence	86
bcfG	Adherence	88
fimY	Adherence	88
safC	Adherence	86
stkA	Adherence	26
stkD	Adherence	26
sthD	Adherence	88
stkE	Adherence	26
stdB	Adherence	86
sthE	Adherence	88
stkC	Adherence	26
fimF	Adherence	88
stfE	Adherence	88
stfD	Adherence	88
safB	Adherence	86
steF	Adherence	26
SeAg_B4897	Adherence	26
stkF	Adherence	26
SNSL254_A4927	Adherence	26
stbE	Adherence	88
lpfD	Adherence	88
stdA	Adherence	86
stkG	Adherence	26
sinH	Adherence	88
misL	Adherence	88
ratB	Adherence	88

Continued

Virulence gene	Gene function	No. of isolated strains
safA	Adherence	24
stcA	Adherence	2
stcB	Adherence	2
stcC	Adherence	2
stcD	Adherence	2
mrkC	Adherence	1
mrkA	Adherence	1
mrkB	Adherence	1
mrkF	Adherence	1
mrkJ	Adherence	1
pegB	Adherence	62
pegC	Adherence	62
faeC	Adherence	26
faeD	Adherence	26
faeE	Adherence	26
pegA	Adherence	62
entA	Antimicrobial activity/Competitive advantage	26
entE	Antimicrobial activity/Competitive advantage	24
mig-14	Antimicrobial activity/Competitive advantage	87
ssrB	Effector delivery system	88
spiC/ssaB	Effector delivery system	88
ssaC	Effector delivery system	88
ssaE	Effector delivery system	88
ssaG	Effector delivery system	88
ssaH	Effector delivery system	88
ssaI	Effector delivery system	88
ssaJ	Effector delivery system	88
ssaN	Effector delivery system	88
ssaR	Effector delivery system	88
ssaS	Effector delivery system	88
ssaU	Effector delivery system	88
invG	Effector delivery system	88
invE	Effector delivery system	88
invA	Effector delivery system	88
invB	Effector delivery system	88
invC	Effector delivery system	88
invI	Effector delivery system	88
spaP	Effector delivery system	88

Continued

Virulence gene	Gene function	No. of isolated strains
spaQ	Effector delivery system	88
spaR	Effector delivery system	88
spaS	Effector delivery system	88
sicA	Effector delivery system	88
iacP	Effector delivery system	88
sicP	Effector delivery system	88
hilA	Effector delivery system	88
hilD	Effector delivery system	88
prgJ	Effector delivery system	88
prgK	Effector delivery system	88
hilC	Effector delivery system	88
sipC	Effector delivery system	88
ssaV	Effector delivery system	88
invJ	Effector delivery system	88
spaO	Effector delivery system	88
sipB	Effector delivery system	88
sprB	Effector delivery system	87
invF	Effector delivery system	88
ssaK	Effector delivery system	88
orgB	Effector delivery system	88
ssaD	Effector delivery system	88
sseB	Effector delivery system	88
orgA	Effector delivery system	88
sseK1	Effector delivery system	87
ssaL	Effector delivery system	88
ssaQ	Effector delivery system	88
sscA	Effector delivery system	88
invH	Effector delivery system	88
ssaO	Effector delivery system	88
sscB	Effector delivery system	88
prgH	Effector delivery system	88
ssaT	Effector delivery system	88
ssaP	Effector delivery system	88
sseA	Effector delivery system	88
sptP	Effector delivery system	88
sipD	Effector delivery system	88
ssrA	Effector delivery system	88
iagB	Effector delivery system	88
sopD	Effector delivery system	87
orgC	Effector delivery system	88
avrA	Effector delivery system	87

Continued

Virulence gene	Gene function	No. of isolated strains
sseE	Effector delivery system	88
sseK2	Effector delivery system	26
sopB/sigD	Effector delivery system	88
ssaM	Effector delivery system	88
sopE2	Effector delivery system	88
sseJ	Effector delivery system	88
sseG	Effector delivery system	88
sipA	Effector delivery system	88
sseF	Effector delivery system	88
pipB	Effector delivery system	88
sopA	Effector delivery system	88
sifB	Effector delivery system	88
prgI	Effector delivery system	88
KOX_00005	Effector delivery system	86
sseC	Effector delivery system	88
slrP	Effector delivery system	88
sseL	Effector delivery system	88
sseD	Effector delivery system	88
sifA	Effector delivery system	88
pipB2	Effector delivery system	87
sciU	Effector delivery system	88
sciR	Effector delivery system	88
sciP	Effector delivery system	88
sciM	Effector delivery system	88
STM0278	Effector delivery system	88
sciK	Effector delivery system	88
sciD	Effector delivery system	88
sciC	Effector delivery system	88
sciI	Effector delivery system	88
sciO	Effector delivery system	88
sciS/icmF-like	Effector delivery system	88
sciA	Effector delivery system	88
sciB	Effector delivery system	88
vgrS	Effector delivery system	86
clpV	Effector delivery system	88
sciV	Effector delivery system	88
sciF	Effector delivery system	88
sciW	Effector delivery system	86
sciE	Effector delivery system	88
sciH	Effector delivery system	88
sciL	Effector delivery system	88

Continued

Virulence gene	Gene function	No. of isolated strains
sciT	Effector delivery system	88
sciN	Effector delivery system	88
sciJ	Effector delivery system	88
sspH2	Effector delivery system	67
sopD2	Effector delivery system	63
fepC	Effector delivery system	64
sseI	Effector delivery system	55
sciQ	Effector delivery system	2
astA	Immune modulation	37
pagN	Immune modulation	88
phoQ	Regulation	88
phoP	Regulation	88
papB	Regulation	3
papI	Regulation	3
rpoS	Regulation	87
fur	Regulation	88
A225_4123	Regulation	88
mgtB	Nutritional/Metabolic factor	88
mgtC	Nutritional/Metabolic factor	88
sodCI	Stress survival	55
grvA	Stress survival	55
