# Supplementary material for: Genomic characterization of Salmonella enterica serovar Kentucky and London recovered from food and human salmonellosis in Zhejiang Province, China (2016–2021)
Source: Front Microbiol. 2022 Aug 4;13:961739. doi: 10.3389/fmicb.2022.961739 (PMC9437622; doi:10.3389/fmicb.2022.961739)
Supplement: Supplementary file 2 [file Data_Sheet_2.pdf]

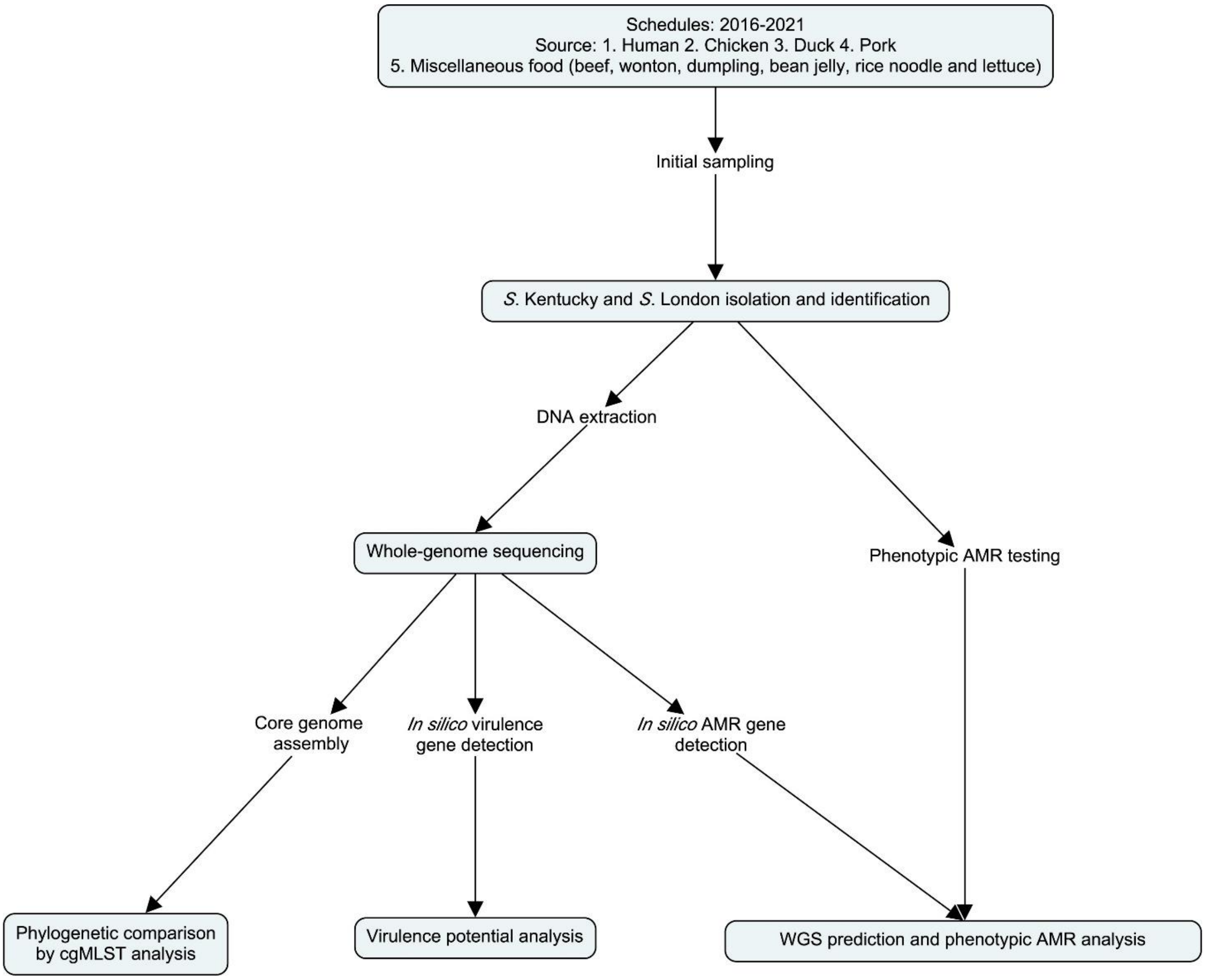

Supplementary Figure S1 Workflow of the procedures employed for genomic analysis of 88 *Salmonella* Kentucky and London strains. The graphic was created by CMapTools (https://cmap.ihmc.us).

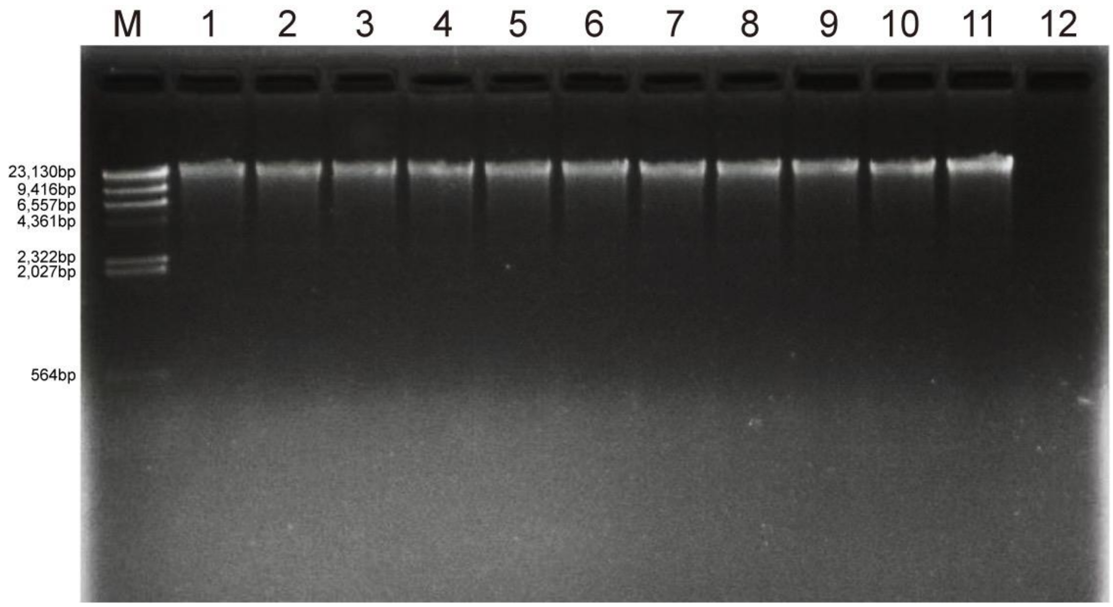

Supplementary Figure S2 *Salmonella* DNA patterns were resolved via gel electrophoresis at 100V for 45 min on 1% agarose gels stained with ethidium bromide. The bands represented total genomic DNA and were visualized with UV transillumination (Bio-Rad). Lanes 1-10: *Salmonella* samples used in this study; Lane 11: positive control *Salmonella* Braenderup H9812; Lane 12: negative control PCR water. M: molecular size marker (TaKaRa, Japan).

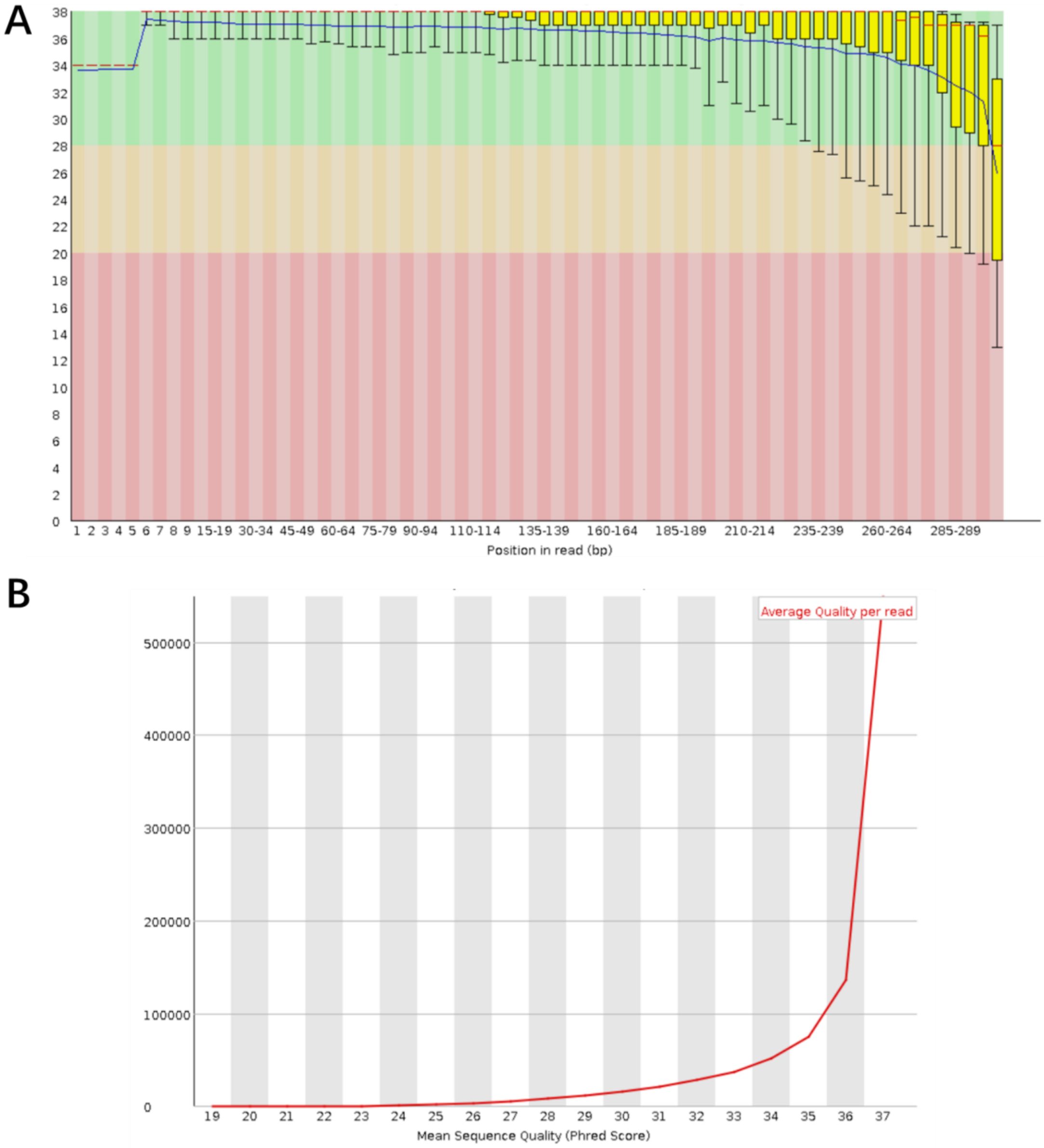

Supplementary Figure S3 Representative quality control reports for **(A)** quality scores across all bases and **(B)** quality score distribution over all sequences.

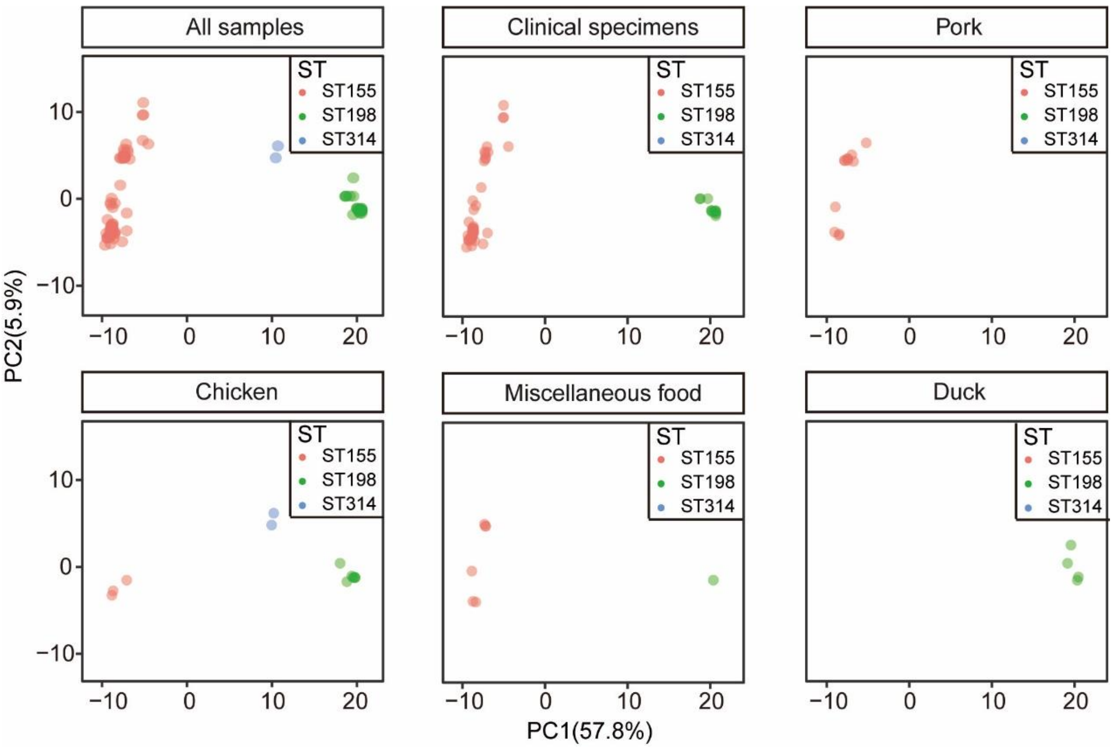

Supplementary Figure S4 PCA-based pan-genome analysis for the differentiation of eighty-eight *Salmonella* enterica serovar Kentucky and London isolates. PCA plot showed ST155 in red, ST198 in green and ST314 in blue.
